# Netrin-5 is highly expressed in neurogenic regions of the adult brain

**DOI:** 10.3389/fncel.2015.00146

**Published:** 2015-04-20

**Authors:** Satoru Yamagishi, Kohei Yamada, Masato Sawada, Suguru Nakano, Norio Mori, Kazunobu Sawamoto, Kohji Sato

**Affiliations:** ^1^Department of Anatomy and Neuroscience, Hamamatsu University School of MedicineHamamatsu, Shizuoka, Japan; ^2^Research Center for Child Mental Development, Hamamatsu University School of MedicineHamamatsu, Shizuoka, Japan; ^3^Department of Developmental and Regenerative Biology, Nagoya City University Graduate School of Medical SciencesNagoya, Japan; ^4^Department of Psychiatry, Hamamatsu University School of MedicineHamamatsu, Shizuoka, Japan

**Keywords:** axon guidance, netrin, adult neurogenesis, rostral migratory stream, subventricular zone, subgranular zone

## Abstract

Mammalian netrin family proteins are involved in targeting of axons, neuronal migration, and angiogenesis and act as repulsive and attractive guidance molecules. Netrin-5 is a new member of the netrin family with homology to the C345C domain of netrin-1. Unlike other netrin proteins, murine netrin-5 consists of two EGF motifs of the laminin V domain (LE) and the C345C domain, but lacks the N-terminal laminin VI domain and one of the three LE motifs. We generated a specific antibody against netrin-5 to investigate its expression pattern in the rodent adult brain. Strong netrin-5 expression was observed in the olfactory bulb (OB), rostral migrate stream (RMS), the subventricular zone (SVZ), and the subgranular zone (SGZ) of the dentate gyrus in the hippocampus, where neurogenesis occurs in the adult brain. In the SVZ and RMS, netrin-5 expression was observed in Mash1-positive transit-amplifying cells and in Doublecortin (DCX)-positive neuroblasts, but not in GFAP-positive astrocytes. In the OB, netrin-5 expression was maintained in neuroblasts, but its level was decreased in NeuN-positive mature neurons. In the hippocampal SGZ, netrin-5 was observed in Mash1-positive cells and in DCX-positive neuroblasts, but not in GFAP-positive astrocytes, suggesting that netrin-5 expression occurs from type 2a to type 3 cells. These data suggest that netrin-5 is produced by both transit-amplifying cells and neuroblasts to control neurogenesis in the adult brain.

## Introduction

Netrin family proteins are diffusible axon guidance molecules. Originally netrin-1 was identified as a chemical attractant for spinal commissural axons during embryonic development (Serafini et al., 1994, 1996). Netrin-1 has a homology to the laminin B2 chain and consists of the N-terminal laminin VI domain, three EGF motifs of the laminin V domain (LE), and the C345C domain. Netrin-1 (Unc-6 in nematodes) is evolutionally well conserved in both vertebrates and invertebrates (Rajasekharan and Kennedy, 2009). Mammals express netrin-1, -3, and -4, whereas netrin-2, the ortholog of netrin-3, exists only in birds and in fish (Wang et al., 1999). The functions of netrin proteins are quite variable. In addition to regulating axonal guidance as both attractive and repulsive cues, netrin proteins promote cell survival by binding to DCC (deleted in colorectal carcinoma) and/or to the Unc5 dependent receptor for both neurons and tumor cells (Arakawa, 2004; Adams and Eichmann, 2010; Castets and Mehlen, 2010). Netrin-1 is also an angiogenic factor that acts as a guidance molecule and promotes proliferation of endothelial cells (Delloye-Bourgeois et al., 2012). Recently, it was reported to promote atherosclerosis by inhibiting the emigration of macrophages from plaques and by attenuating hypoxia-elicited inflammation at mucosal surfaces (Ramkhelawon et al., 2014).

Neurogenesis in the adult brain occurs throughout life at two major locations: the subventricular zone (SVZ) of the lateral ventricles and the subgranular zone (SGZ) of the dentate gyrus in the hippocampus (Zhao et al., 2008). In the anterior of the SVZ, neuroblasts, derived from progenitor cells, form chains and migrate along a restricted route, referred to as the rostral migrate stream (RMS). During the migration, the stabilization, and destabilization of the microtubules in the neuroblasts are regulated by DCX and stathmin1, respectively. Disruption of either DCX or stathmin1 disturbed the chain migration in the RMS (Camoletto et al., 1997; Jin et al., 2010). Subsequently neuroblasts terminate migration and localize in the olfactory bulb (OB), where they differentiate into interneurons (Doetsch et al., 1999; Alvarez-Buylla and Garcıa-Verdugo, 2002). The chains of neuroblasts migrate through a tunnel of GFAP-positive astrocytes called the “glial tube” which are derived from type B cells. During the migration, neuroblasts are guided by various molecules. Soluble netrin-1 is secreted from the mitral cells in the OB to attract DCC-positive neuroblasts (Murase and Horwitz, 2002). Inhibiting DCC by an antibody disrupted the direction of the migrating chain of cells. Another netrin-1 receptor, neogenin, is known to be expressed in both neuroblasts and GFAP-positive astrocytes in the RMS of both rodents and humans (Bradford et al., 2010). Neural stem cells in another region of the brain, the SGZ in the adult hippocampus, can produce intermediate progenitors (type 2a cells), which in turn generate neuroblasts (type 2b to type 3 cells) (Eriksson et al., 1998). Recently, it was shown that DCX-positive type 2b cells can also proliferate and expand the precursor pool (Lugert et al., 2012). After exiting the cell cycle, these cells differentiate into granule neurons. Although much is known about adult neurogenesis in the SGZ, nothing is known about the contribution of netrins and their receptors to neurogenesis.

We previously characterized new functions of FLRT2/3 as novel repulsive axon guidance molecules acting via Unc5s (Yamagishi et al., 2011). In order to identify additional axon guidance molecules, we performed BLAST analyses to search for proteins with sequences homologs to netrin-1. Here, we report an uncharacterized protein, netrin-5, found on the NCBI database, which was previously named due to its homology to other netrin family proteins. We show that netrin-5 is strongly expressed in neuroproliferative regions, namely in the SVZ and SGZ. Netrin-5 is co-expressed with Mash1, DCX, and stathmin1, which regulates microtubule stability, in neuroblasts in both the SVZ and RMS, whereas GFAP-positive cells do not co-express netrin-5. Consistent with these findings, Mash1-positive cells and DCX-positive neuroblasts in the SGZ also co-expressed netrin-5, indicating netrin-5 expression occurs from type 2a to type 3 cells. These expression patterns suggest that netrin-5 plays a role in adult neurogenesis.

## Materials and methods

### Animals

Wister rats (Japan SLC Inc.) aged 2–3 months were used in most of the experiments unless otherwise stated. Two month old C57BL/6 mice (Japan SLC Inc.) were used for anti-CD31 (PECAM1) antibody staining. Glial fibrillary acidic protein (GFAP) expression was analyzed using 2 month old transgenic mice overexpressing GFP under control of the astrocyte-specific GFAP promoter (*Gfap-EGFP* mice, Mutant Mouse Regional Resource Center; Kaneko et al., 2010). All animal experiments were approved by the Committee on Animal Research at Hamamatsu University School of Medicine and performed according to the national guidelines and regulations in Japan.

### Antibodies

To generate polyclonal antibodies, rabbits were immunized once and boosted 3 times with a synthetic peptide (C+FKQRAWPVRRGGQE; 353-366 aa) corresponding to the sequence of the C345C domain of mouse netrin-5a (NP_001028528.2). Antibodies were purified from serum using an antigen-specific affinity column (Operon Biotech.). The antigen sequence was chosen by the antigen prediction program (Operon Biotech). The following commercial antibodies were also used for immunofluorescence and western blotting: anti-GAPDH (1:5000, mouse monoclonal, Abcam, ab8245), anti-DCX (1:500, goat polyclonal, Santa Cruz, sc-8066), anti-stathmin1 (1:500, mouse monoclonal, GeneTex, GTX62235), anti-Mash1 (1:500, mouse monoclonal, BD, #5566045), anti-GFAP (1:1000, mouse monoclonal, Millipore, MAB3402), anti-GFP (JL-8; 1:500, mouse monoclonal, Clontech, 632381), anti-NeuN (1:100, mouse monoclonal, Millipore, MAB377), and anti-CD31 (PECAM1, 1:100, rat monoclonal, BD, #550274) antibodies.

### Histological analysis

The animals were deeply anesthetized and intracardially perfused with 4% paraformaldehyde (PFA)/PBS for 5 min. Brains were dissected, post fixed in 4% PFA/PBS for 10 min, and frozen at −80°C. Cryostat sagittal sections at 20 μm thickness were fixed in 4% PFA/PBS for 10 min. For anti-Mash1 antibody staining, 50 μm thick vibratome sections were cut. Endogenous peroxidase was quenched in 0.3% H_2_O_2_ in methanol for 20 min at RT. After washing with PBS, the sections were permeabilized in 0.3% Triton-X 100/PBS for 3 min. Then, the sections were incubated with blocking solution containing 3% normal goat serum (NGS) in 0.1% Triton-X 100/PBS for 1 h at RT followed by incubation of primary antibodies in 1% NGS/0.1% Triton-X 100/PBS overnight at 4°C. Either HRP-conjugated (DAKO) or Alexa Fluor dye-conjugated secondary antibodies (Life Technologies) were incubated with the sections for 30 min at RT. For anti-Mash1 antibody staining, tyramide Signal Amplification (TSA) amplification was performed according to manufacturer's instructions (PerkinElmer, NEL701001KT). The sections treated with HRP-conjugated antibody were visualized by ImmPACT DAB (DAKO) according to the manufacturer's instructions. The samples from the *Gfap-EGFP* mice were treated with anti-GFP (JL-8) antibody to amplify the GFP signal. The sections with fluorescent secondary antibodies were observed using confocal microscopy FV1000 (Olympus). Similar results were obtained from at least three independent animals.

### Western blotting

Total cell lysates from different brain regions were prepared in lysis buffer, consisting of 1% Triton X-100/TBS supplemented with a proteinase inhibitor cocktail (Roche). The samples from three rats were pooled and resolved on a SDS-PAGE gel. Then, the proteins were transferred to a PVDF membrane and the membrane was incubated sequentially with blocking solution, primary antibody, and HRP-conjugated secondary antibody. The membrane was developed with the enhanced chemiluminescence (ECL) reagent (Thermo Scientific).

## Results

### Netrin-5 homology to netrin-1

**Figure 1:**
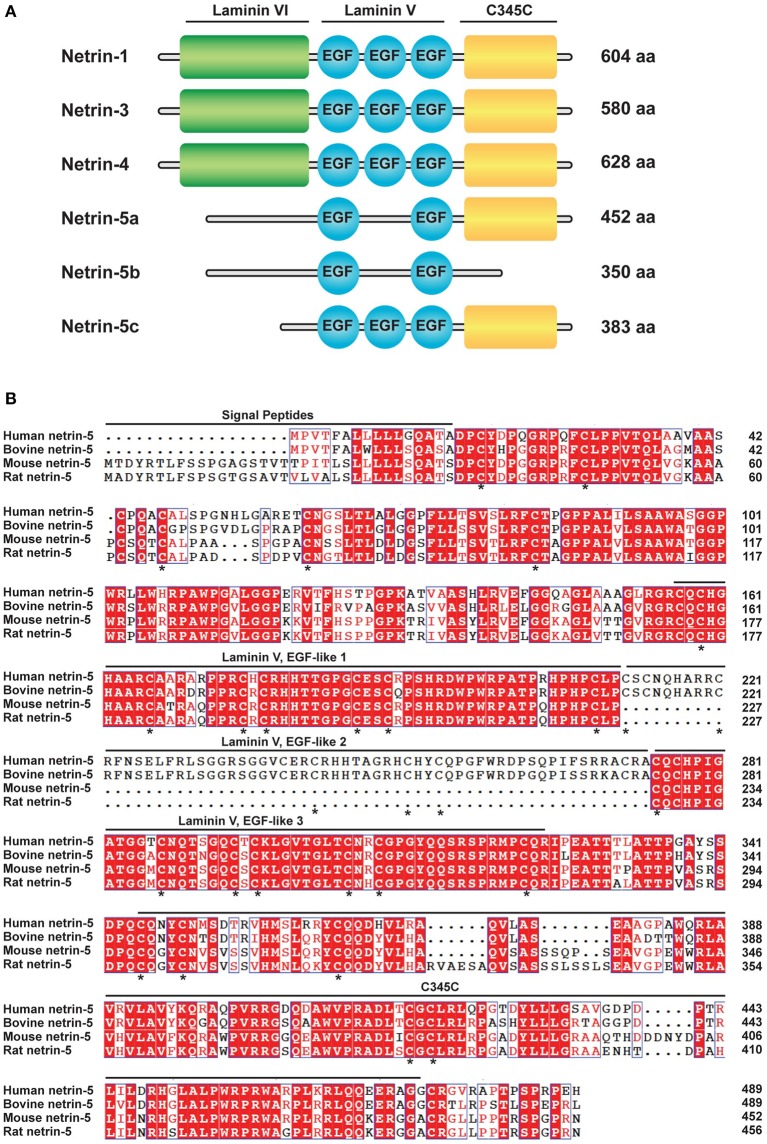
**Netrin-5, a new member of the netrin protein family.(A)** Schematic drawing of the domain structure of the netrin family (*Mus musculus*; NTN1, 3, 4, and 5a-c). NTN5a and NTN5b lack the laminin VI domain and one of EGF-like motifs of the laminin V domain. NTN5b further lacks the C345C domain. NTN5c is smaller than NTN5a but contains three EGF-like domains. (**B**) Alignment of the predicted amino acid sequences of Homo sapiens NTN5 (accession number NP_665806.1), Bos taurus NTN5 (NP_001193801.1), *Mus musculus* NTN5 isoform a (NP_001028528.2), and *Rattus norvegicus* NTN5 (NP_001166997.1). Amino acid residues identical among the species are shaded in red. Similar residues are colored in red but not shaded. Putative signal peptides, EGF-like motifs of the laminin V domain, and the C345C domain are indicated. An asterisk (*) indicates conserved cysteine in netrin-1 and in netrin-5.

In order to search for a new axon guidance protein homologs to murine netrin-1, we performed protein BLAST analysis with the mouse netrin-1 C345C domain (472-601 aa), which is partially involved in binding to the Unc5 receptor (Kruger et al., 2004). As a result, netrin-5 isoform c (accession no. NP_001276622; 383 aa) showed the highest score with 32% identity to netrin-1. Unlike the structures of netrins-1, -3, and -4, full length netrin-5a (NP_001028528.2) consists of 452 aa comprising two laminin V-type EGF-like (LE) domains and the C345C domain, but lacking the N-terminal laminin VI domain (Figure 1A). A splice variant, netrin-5b, has a unique short tail at the C-terminus instead of the C345C domain. Netrin-5c is shorter than this isoform because it lacks the N-terminal sequence but contains 3 LE domains. Netrin-5s from both human and bovine are different from rodent netrin-5. Human and bovine netrin-5 has a shorter signal peptide but contains all three LE domains (Figure 1B). Many amino acids in both the LE1 and LE3 domains are highly conserved among these 4 species. Homology between human and mouse netrin-5 is 73%. Interestingly, most cysteine residues are highly conserved between netrin-1 and netrin-5, suggesting that the 3D structure of netrin-5 might be similar to that of netrin-1. Netrin-5 exists only in mammals, suggesting that it was evolutionarily acquired later than the other netrin family proteins.

### Netrin-5 expression in the adult brain

**Figure 2:**
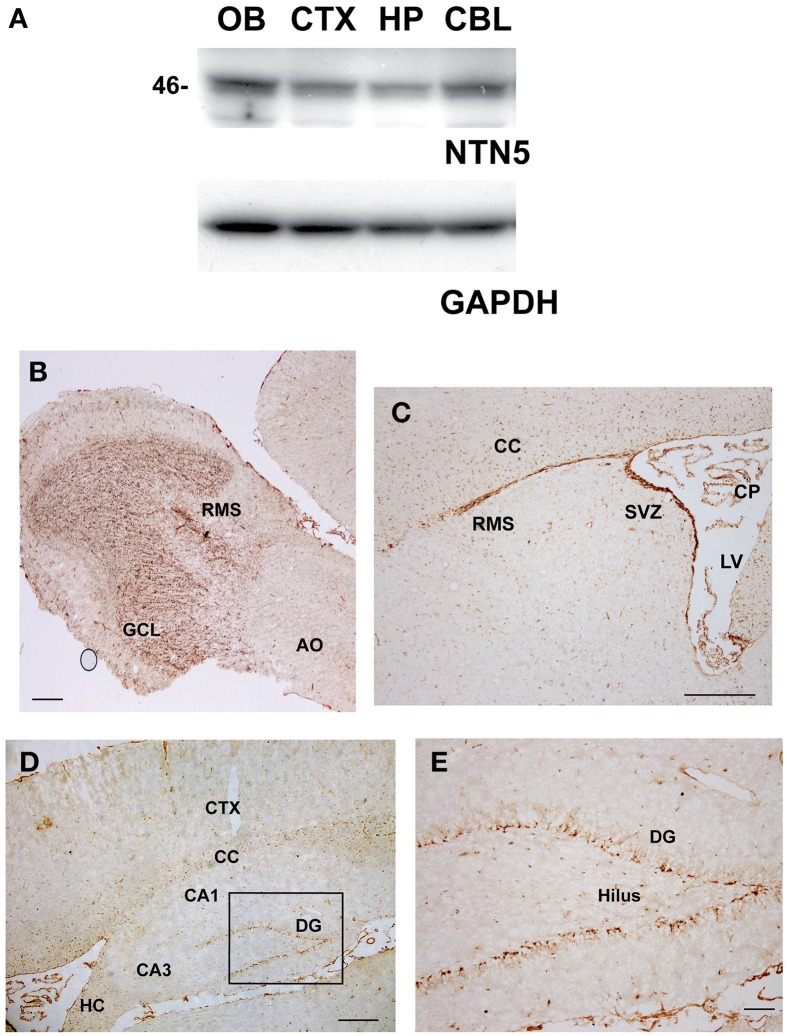
**Expression of netrin-5 in the adult brain. (A)** Immunoblotting analysis revealed that anti-netrin-5 antibody recognizes a protein of ~46 kDa in the total cell lysate of the dissected adult rat brains. Netrin-5 expression was higher in the olfactory bulb and lower in the hippocampus. The same membrane was reprobed with anti-GAPDH antibody as a loading control (lower panel). **(B–E)** Immunohistochemistry of sagittal sections of adult rat brain with anti-netrin-5 antibody. Anti-netrin-5 antibody stained strongly in the GCL of the OB **(B)**, SVZ, RMS, CP **(C)**, and SGZ of the DG **(D,E)**. AO, anterior olfactory nucleus; CBL, cerebellum; CC, corpus callosum; CP, choroid plexus; CTX, cerebral cortex; DG, dentate gyrus; GCL, granule cell layer; HP, hippocampus; OB, olfactory bulb; RMS, rostral migratory stream; SGZ, subgranular zone; SVZ, subventricular zone. Bars indicate 500 μm in **(B–D)**, and 100 μm in **(E)**.

In order to explore the expression pattern of netrin-5 in adult rat brain, we generated an antibody against the C345C domain. The level of netrin-5 protein in the adult rat brain was first evaluated by immunodetection on western blotting using a rabbit polyclonal antibody against the C345C domain. Netrin-5 appeared at ~46 kDa and is expressed in the OB, the cerebral cortex, the hippocampus, and the cerebellum in the adult brain (Figure 2A). In the OB, the expression level was relatively higher than that in other regions. In the hippocampus, netrin-5 showed a weak expression level. Consistent with the western blotting, immunohistochemistry on sagittal sections showed extensive expression in the OB (Figure 2B). Netrin-5 immunoreactivity was observed throughout the granule cell layer of the OB. In the rostral migratory stream (RMS), the immunoreactivity was strong. However, a weaker signal in the outer plexiform layer and almost no signal were observed in the anterior olfactory nucleus. In accordance with the strong immunoreactivity in the RMS of the OB, strong netrin-5 expression was also observed throughout the RMS from the anterior wall of the SVZ of the lateral ventricle (Figure 2C). In addition, the choroid plexus had a positive signal. Non-clustered netrin-5 positive signal was observed in both the corpus callosum and hippocampal commissure (Figures 2C,D). Sparse immunoreactivity was observed in both the striatum and the cerebral cortex. These cells were positive for CD31, a vascular endothelial marker (Figure S1). Only a subset of endothelial cells showed netrin-5-immunoreactivity. Since netrin-5 is strongly expressed in both the SVZ and RMS, it may play a role in adult neurogenesis. Therefore, we next focused on the subgranular zone (SGZ) of the dentate gyrus in the hippocampus, the other neurogenic region in the adult brain. Although the immunoreactivity in the hippocampus was relatively low by western blotting, netrin-5 signal was specifically observed in the SGZ as expected (Figures 2D,E). There were few positive cells in the pyramidal cell layer of both the CA1 and CA3 regions and in the hilus region.

### Netrin-5 expression in transit-amplifying cells (type C cells) and neuroblasts (type A cells), but not in neural stem cells (type B cells), in the SVZ

**Figure 3:**
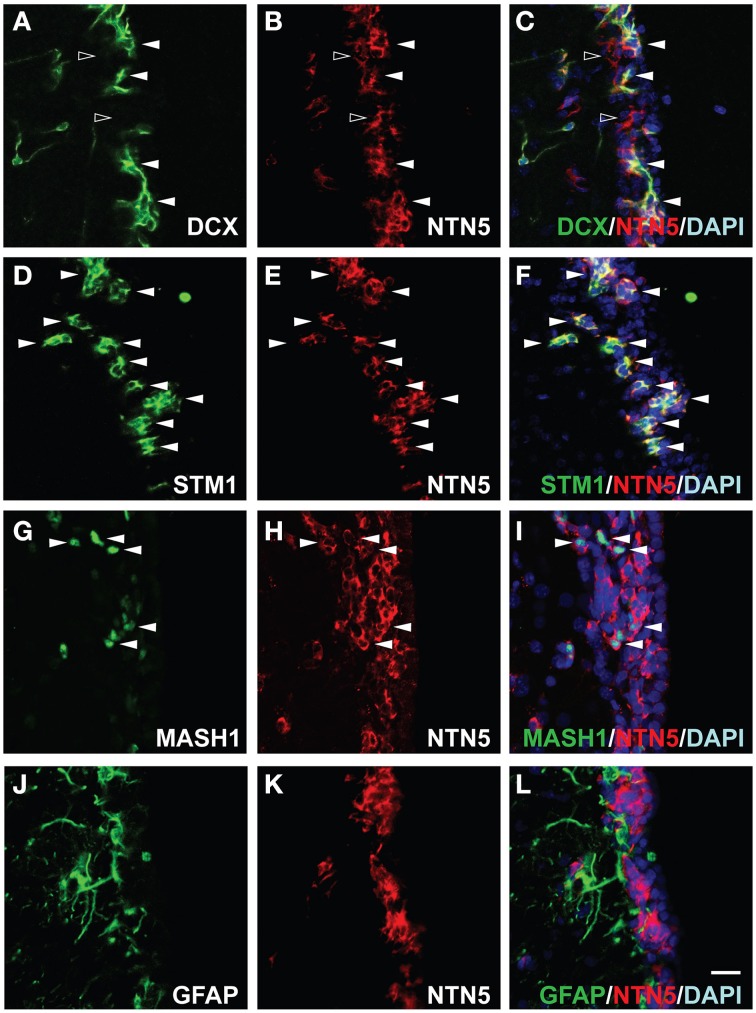
**Expression of netrin-5 in the SVZ of adult rat brain. (A–C)** Immunostaining of sagittal sections of adult rat brain revealed that DCX-positive cells in the SVZ stained for netrin-5 as observed in confocal imaging (white arrowheads). Some netrin-5-positive cells were DCX-negative (black arrowheads). (**D–F**) Stathmin1 and netrin-5 showed high co-localization (white arrowheads). (**G–I**) Mash1-positive cells are also netrin-5-positive (white arrowheads). (**J–L**) Netrin-5 expression did not co-localize with GFAP. Bar indicates 50 μm.

In the adult SVZ, a subset of the astrocyte-like cells (type B cells) are proliferative stem cells that generate neuroblasts (type A cells) via transit-amplifying cells (type C cells). The majority of netrin-5 immunoreactive cells in the SVZ showed a morphology typical of the chain-forming neuroblasts. In order to determine the netrin-5-expressing cell types in the SVZ, we performed double-immunolabeling in a sagittal section of the rat adult brain using antibodies against netrin-5 and DCX, a microtubule binding protein widely used as a marker for neuroblasts (Zhao et al., 2008). As shown in Figures 3A–C, all DCX-positive cells at the SVZ co-expressed netrin-5. There were some netrin-5-positive SVZ cells that did not co-express DCX, suggesting that netrin-5 expression is initiated in DCX-negative type C cells. We also stained for the microtubule binding protein stathmin1, which is expressed in the SVZ cells, including neuroblasts (Camoletto et al., 1997; Jin et al., 2004). Double immunostaining showed complete co-localization of stathmin1 and netrin-5 (Figures 3D–F). Since stathmin1 expression is regulated by Mash1 (Ascl1) (Yamada et al., 2010), we then evaluated netrin-5 expression in Mash1-positive transit-amplifying cells (Figures 3G–I). As a result, all Mash1 positive cells were netrin-5 positive, indicating that transit-amplifying cells also express netrin-5 (Figures 3G–I). Next, we determined whether netrin-5 expression occurs in type B cells. Netrin-5 was not detected in GFAP-positive cells (type-B cells or other astrocytes; Figures 3J–L). Taken together, these results suggest that netrin-5 is expressed in transit-amplifying cells and in neuroblasts but not in either neural stem cells or astrocytes in the SVZ.

### Netrin-5 expression in neuroblasts in the RMS

**Figure 4:**
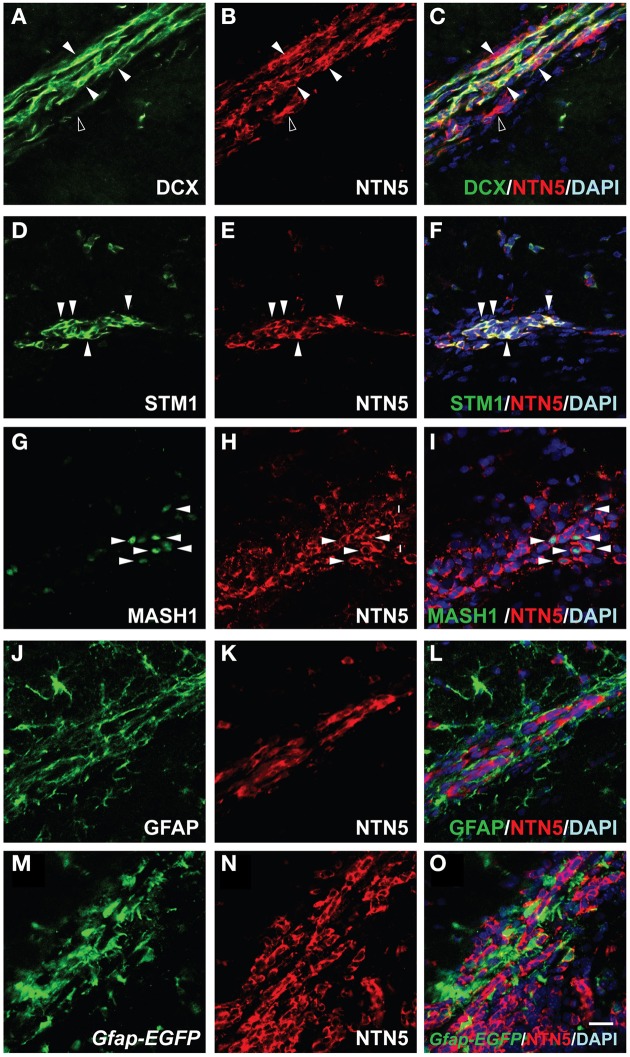
**Netrin-5 expression by neuroblasts and transit-amplifying cells but not by astrocytes within the RMS. (A–C)** Immunostaining of sagittal sections of rat adult brain showed that most of the DCX-positive cells in the RMS stained for netrin-5 as observed in confocal imaging (white arrowheads). Some netrin-5-positive cells were DCX-negative (black arrowhead). (**D–F**) Stathmin1 and netrin-5 showed high co-localization (white arrowheads). (**G–I**) Anti-Mash1 staining revealed that Mash1-positive cells express netrin-5 (white arrowheads). (**J–L**) Anti-GFAP staining showed that netrin-5 was not expressed in astrocytes. (**M–O**) Most *Gfap-EGFP* cells did not show netrin-5 expression. (**A–L**) adult rat brain and (**M–O**) adult *Gfap-EGFP* mouse brain. Bar indicates 50 μm.

Since the newly formed neuroblasts in the SVZ migrate toward the anterior region through the RMS, we next characterized the netrin-5-positive cells in the RMS. Consistent with the result in the SVZ, we found DCX-positive cells highly co-localized with the netrin-5 signal in the RMS (Figures 4A–C). In contrast to the homogeneous expression of DCX in the neuroblasts, netrin-5 was expressed at various levels in the cells. There were some netrin-5-positive cells that did not co-express DCX. On the other hand, almost all of the netrin-5-positive cells showed co-localization with stathmin1 (Figures 4D–F). Mash1-positive transit-amplifying cells were also positive for netrin-5 (Figures 4G–I), suggesting that the DCX-negative- and netrin-5-positive cells (Figures 4A–C) were transit-amplifying cells. The chains of rapidly migrating neuroblasts are ensheathed by a meshwork of GFAP-positive astrocytes, namely the “glial tube” (Lois and Alvarez-Buylla, 1994; Lois et al., 1996; Kaneko et al., 2010). Although GFAP and netrin-5 immunoreactive signals are spatially intermingled, none of the GFAP-positive signal co-localized with netrin-5 (Figures 4J–L). Since GFAP is a cytoskeletal protein and anti-GFAP antibody staining cannot visualize the entire shape of the astrocytes, we utilized GFAP promoter-controlled EGFP transgenic mice (*Gfap-EGFP*). As expected, most of netrin-5-positive cells are GFP-negative, confirming the mutually exclusive expression of netrin-5 and GFAP (Figures 4M–O).

### Netrin-5 expression in both neuroblasts and neurons in the GCL of the OB

**Figure 5:**
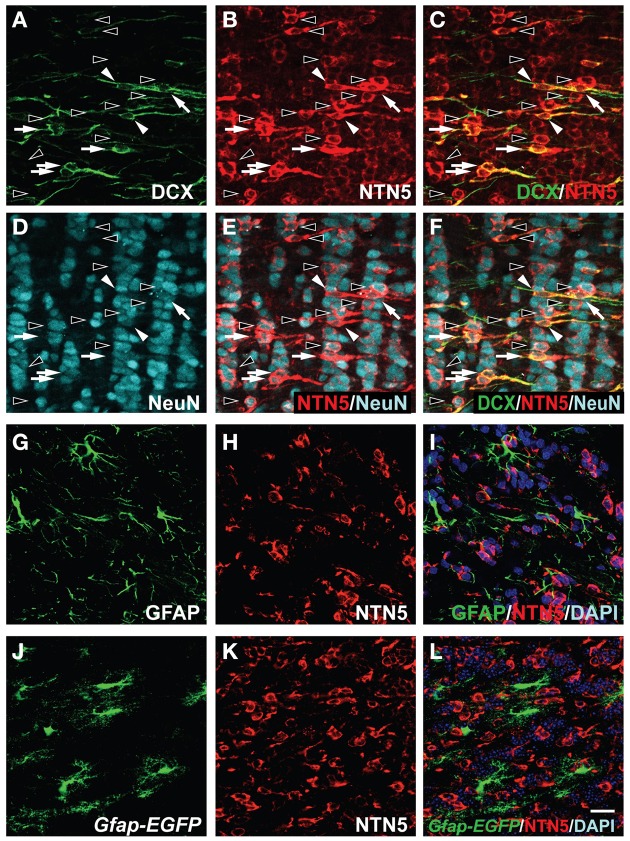
**Expression of netrin-5 in the GCL of the OB in the adult brain. (A–F)** Triple-immunostaining of sagittal sections of adult rat brain using anti-DCX, anti-netrin-5, and anti-NeuN antibodies. Netrin-5 was highly expressed in Dcx+ neuroblasts (white arrowheads), Dcx+NeuN+ immature neurons (white arrows), and a subset of NeuN+ mature neurons (black arrowheads), and decreased but still detectable in most NeuN+ mature neurons. (**G–I**) Anti-GFAP staining showed that netrin-5 was not expressed in astrocytes. (**J–L**) *Gfap-EGFP* cells did not show netrin-5 expression. (**A–I**) adult rat brain and (**J–L**) adult *Gfap-EGFP* mouse brain. Bar indicates 50 μm.

The migrated neuroblasts are differentiated into GABAergic neurons and integrated in the GCL of the OB (Abrous et al., 2005). Since the strong immunoreactivity of netrin-5 was observed in the GCL (Figure 2B), we attempted to determine the cell type in the GCL. The netrin-5 signal was observed in the cell body and in the basal part of the apical dendrites of the DCX-positive new neurons (Figures 5A–C). Since only about 20% of the netrin-5-positive cells in the GCL co-expressed DCX, we next analyzed whether netrin-5 expression is present in mature neurons. As expected, the DCX-negative- and netrin-5-positive cells were NeuN-positive, indicating that netrin-5 is also expressed in mature GCs (Figures 5D–F). The expression level of Netrin-5 is stronger in the DCX-positive cells than that in the NeuN-positive cells, suggesting that netrin-5 expression is decreased during the maturation process. Consistent with the exclusive expression of GFAP and netrin-5 in both the SVZ and RMS, we could not observe co-localization of GFAP and netrin-5 in the GCL of the OB (Figures 5G–L), indicating that netrin-5 is not expressed by astrocytes.

### Netrin-5 expression in type 2a to type 3 cells in the SGZ of the hippocampal dentate gyrus

**Figure 6:**
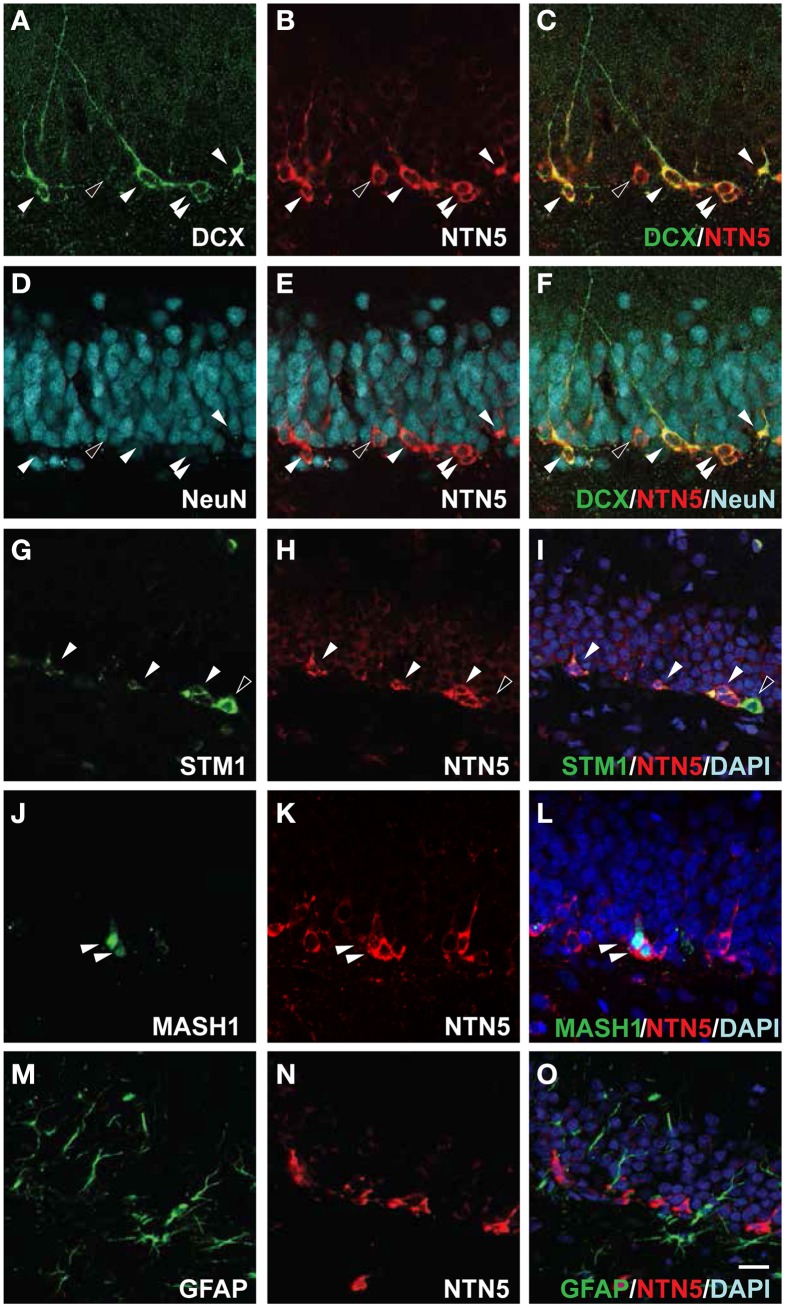
**Netrin-5 expression by type 2 a to type 3 cells but not by type 1 cells within the SGZ. (A–F)** Triple-immunostaining of sagittal sections of adult rat brain with anti-DCX, anti-netrin-5, and anti-NeuN antibodies. DCX-positive cells in the SGZ were stained for netrin-5 as observed in confocal imaging (white arrowheads). Small populations of netrin-5-positive cells were DCX-negative, NeuN-low (black arrowhead). Please note that most of NeuN-positive cells were netrin-5 negative. (**G–I**) Stathmin1 and netrin-5 showed partial co-localization (white arrowheads). (**J–L**) Anti-Mash1 staining revealed that Mash1-positive cells express netrin-5 (white arrowheads). (**M–O**) Anti-GFAP staining showed that netrin-5 was not expressed in astrocytes. Bar indicates 50 μm.

Since DCX-positive cells showed netrin-5 immunoreactivity in the SVZ, RMS, and the GCL of the OB, we next analyzed the other major location of adult neurogenesis in rat brain, the subgranular zone (SGZ) of the hippocampal dentate gyrus (Eriksson et al., 1998). As shown in Figures 6A–C, all DCX-positive cells showed co-localization with the netrin-5 signal. In the same field, anti-NeuN staining revealed that most of netrin-5-positive cells contain undetectable or very low levels of NeuN (Figures 6D–F). This was in contrast to the OB where netrin-5 expression remained in mature neurons (Figures 5D–F). Most of netrin-5-positive cells also contain stathmin1 (Figures 6G–I), similar to that in both the SVZ and RMS. Next, we analyzed the expression of Mash1 in netrin-5-expressing cells. Consistent with the observation in both the SVZ and RMS, netrin-5 was also observed in Mash1-positive transient-amplifying cells (Figures 6J–L). Finally, we analyzed netrin-5 expression in neural stem cells (type 1 cells). Double-immunostaining for both GFAP and netrin-5 revealed that the GFAP-positive signal did not co-localize with netrin-5 (Figures 6M–O). Consistent with this result, none of the GFAP promoter-derived GFP-positive cells merged with netrin-5-positive cells (data not shown). Taken together, these results indicate netrin-5 is expressed from type-2a through to type-3 cells during adult neurogenesis in the hippocampus.

## Discussion

Here, we have characterized a new netrin family member, netrin-5, which is expressed in neuroproliferative zones and is associated with migratory pathways in the adult brain, i.e., the SVZ, RMS, OB, and SGZ. It is still unclear whether netrin-5 is a secreted or a cytosolic protein. According to the computational prediction based on the databases, netrin-5 has a putative 34 amino acids signal peptide on the N-terminus for secretion. In addition, 11 disulfide bonds and N-glycosylation sites are suggested. Given that netrin-5 is secreted, it is uncertain which receptor(s) it recognizes. Netrin-1 and -3 bind to DCC, Unc5s, DSCAM, and Neogenin (Keino-Masu et al., 1996; Wang et al., 1999; Ly et al., 2008; Lai Wing Sun et al., 2011). Netrin-1 shows higher affinity to DCC than that of netrin-3 (Wang et al., 1999). Netrin-4 does not bind to the DCC receptor but binds to various integrin family proteins, i.e., integrin α6β1, α6β4, α2β1, and α3β1 (Staquicini et al., 2009; Larrieu-Lahargue et al., 2011; Yebra et al., 2011; Hu et al., 2012). The binding of netrin-4/integrin is mediated by the unique N-terminal laminin VI domain of netrin-4, which is not conserved in netrin-1 and −3. Since netrin-5 is lacking this laminin VI domain, it may not bind to DCC, Unc5s, and integrins. On the other hand, the deletion of the laminin VI domain of netrin-1 did not affect the binding to DCC (Kruger et al., 2004). Interestingly, three binding sites on the netrin-1/DCC complex have been identified by crystal structure analysis (Finci et al., 2014; Xu et al., 2014). Among those binding sites, the laminin VI domain and LE3 domain of netrin-1 bind to DCC FN5 and FN4 domains, respectively, resulting in a continuous netrin-1/DCC assembly as proposed by Xu et al. (2014). Furthermore, Kruger et al. (2004) mapped netrin-1/Unc5c binding sites and found multiple binding sites on netrin-1, including the C345C domain. Therefore, the LE3 domain and C345C domain of netrin-5 might bind to DCC and to Unc5s, respectively. Those binding analyses should be performed in the future to identify the receptors for netrin-5.

Interestingly, netrin-5 is strongly expressed in the SVZ, RMS, and SGZ of the DG in the hippocampus where neurogenesis occurs in the adult brain (Figures 2B–D). Netrin-5 is also expressed in the choroid plexus, where netrin-1 is also expressed (Lein et al., 2007), and in both the corpus callosum and the hippocampal commissure. In the RMS, a major population of netrin-5-positive cells express Mash1, DCX, and stathmin1, namely in transit-amplifying cells and in neuroblasts, but it is not expressed in the GFAP-positive astrocytes, suggesting the involvement of netrin-5 in neurogenesis. Consistently, netrin-5 expression is decreased during neuronal maturation in both the GCL and the DG. On the other hand, NeuN-positive neurons also express weak levels of netrin-5 in the GCL, suggesting that netrin-5 might have a function during the maturation process, such as dendritogenesis and synaptogenesis in GABAergic neurons.

The netrin-1 receptors, DCC and neogenin, are also expressed in neuroblasts in both the RMS and SVZ (Murase and Horwitz, 2002; Bradford et al., 2010). In contrast to netrin-5, Netrin-1 is not detected in the RMS but is located on the outer border of the most ventral end of the descending limb of the RMS and in mitral cells in the OB (Bradford et al., 2010). Inhibition of DCC function by a blocking antibody disturbs the direction of neuroblast migration and reduces the speed of migration (Murase and Horwitz, 2002). Therefore, we hypothesize that this DCC-dependent migration may be also mediated by netrin-5 acting either in an autocrine or paracrine manner. DCC is also known as a dependent receptor, meaning that DCC-positive cells undergo apoptosis without their ligand. Since netrin-1 expression is limited in the anterior region of the RMS, another ligand is needed to maintain the survival of the neuroblasts. We hypothesize that netrin-5 binds to DCC to prevent cell death. In order to clarify the molecular function of netrin-5 as a guidance molecule, *in vitro* analysis, such as binding assays to define receptors, a turning assay, and a growth cone collapse assay are necessary. In addition, loss and/or gain of function studies using animal models will be needed to better understand the *in vivo* functions of netrin-5.

Netrin-1 is well characterized as a repulsive guidance cue during angiogenesis via the Unc5B receptor, which is expressed in arterial endothelial cells, sprouting capillaries, and tip cells (Lu et al., 2004). UncB activation by Netrin-1 causes retraction of the filopodia of the endothelial tip cells and inhibits the neovessel sprouting processes. Since netrin-5 expression was observed in a subpopulation of CD31-positive endothelial cells, netrin-5 might have a role in guiding angiogenesis as a repulsive guidance cue acting either in an autocrine or paracrine manner via Unc5B.

A number of studies revealed that cognition and adult neurogenesis are highly correlated in rodents (Mu and Gage, 2011). Indeed, many mouse models of Alzheimer's disease (AD), such as Presenilin-1 with both M146V and ΔExon9 mutations in knock-in mice and transgenic mice with the Swedish APP mutation, showed decreased adult neurogenesis (Wang et al., 2004; Zhang et al., 2007; Choi et al., 2008). Even in humans, substantial neurogenesis occurs in the hippocampus of the adult brain (Spalding et al., 2013). Although it is still controversial whether the proliferative ratio of neural stem cells is altered, neuronal maturation is impaired in the DG of AD patients (Li et al., 2008). Recently, it has been reported that new neurons are generated in the adult human striatum, which is impaired in patients with Huntington's disease (Ernst et al., 2014). Therefore, it would be of interest to investigate netrin-5 expression in the neurogenic regions in the human brain especially in patients with neurodegenerative disorders.

Although it is widely accepted that new neurons are continuously generated and incorporated into the functional neural network of the adult brain, the neurogenic cues, and the soluble factors involved in the proper transportation of neuroblasts to their appropriate location are still unclear. Our finding of the expression of netrin-5 in neurogenic regions may provide some hints toward understanding the fundamental processes (proliferation, migration, and differentiation) in adult neurogenesis.

### Conflict of interest statement

The authors declare that the research was conducted in the absence of any commercial or financial relationships that could be construed as a potential conflict of interest.

## Supplementary material

Figure S1 **Netrin-5 is expressed in vascular endothelial cells in both the cerebral cortex and the striatum. (A–F)** Immunostaining of sagittal sections of adult rat brain with anti-CD31 and anti-netrin-5 antibodies in the cerebral cortex (**A–C**) and in the striatum (**D–F**). Please note that netrin-5 is expressed in a small population of vascular endothelial cells. White and black arrowheads indicate co-localization and non-co-localization, respectively. Bar indicates 50 μm **(A–F)**.

## Abbreviations

AD: Alzheimer's disease
DCC: deleted in colorectal carcinomas
DCX: doublecortin
DG: dentate gyrus
GCL: granule cell layer
GFAP: glial fibrillary acidic protein
LE: EGF motifs of laminin V domain
OB: olfactory bulb
RMS: rostral migratory stream
SVZ: subventricular zone
SGZ: subgranular zone.
