# Supplementary figures and images for: Netrin-5 is highly expressed in neurogenic regions of the adult brain
Source: Front Cell Neurosci. 2015 Apr 20;9:146. doi: 10.3389/fncel.2015.00146 (PMC4403520; doi:10.3389/fncel.2015.00146)

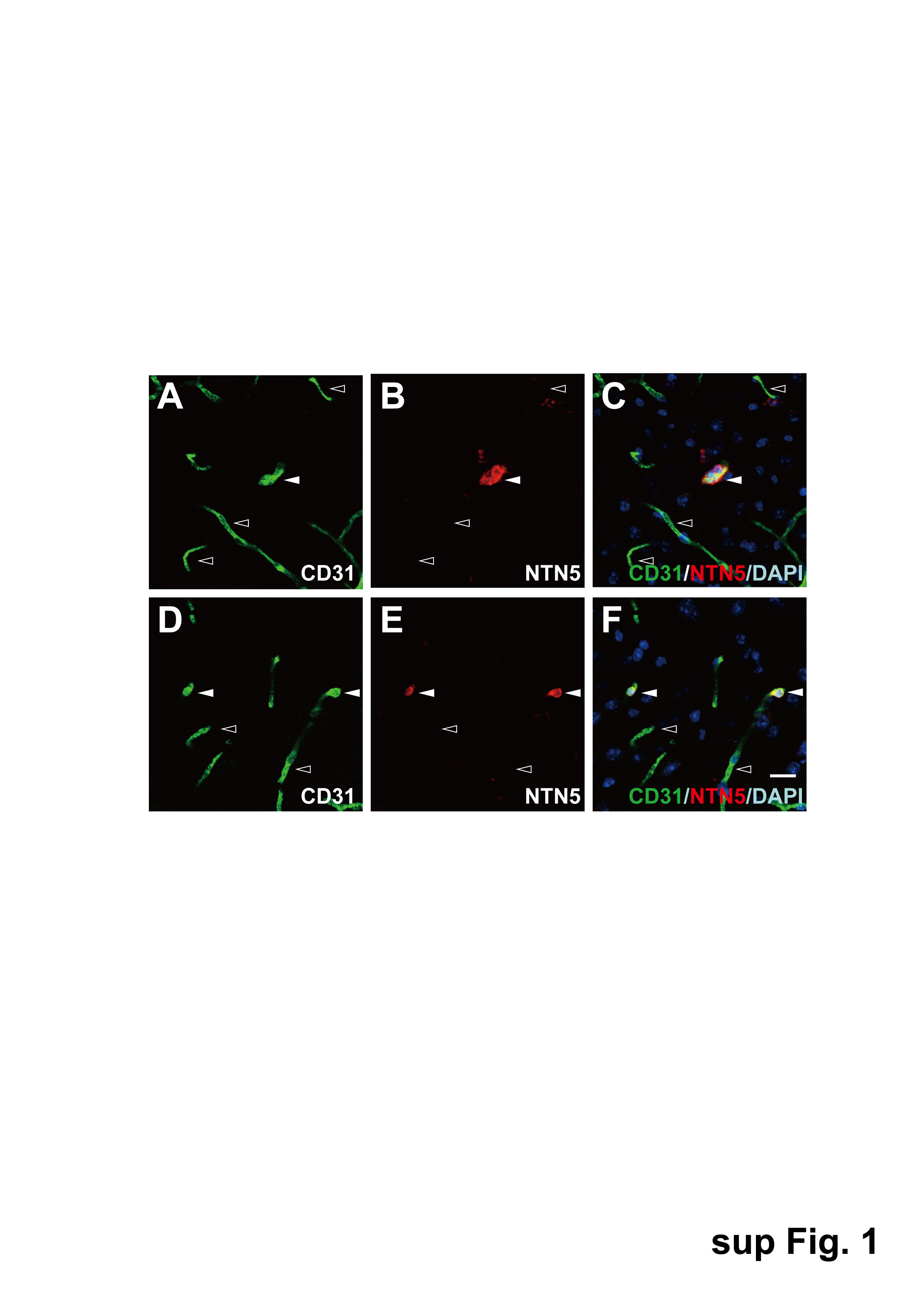

Supplement: Figure S1 — Netrin-5 is expressed in vascular endothelial cells in both the cerebral cortex and the striatum. (A–F) Immunostaining of sagittal sections of adult rat brain with anti-CD31 and anti-netrin-5 antibodies in the cerebral cortex (A–C) and in the striatum (D–F). Please note that netrin-5 is expressed in a small population of vascular endothelial cells. White and black arrowheads indicate co-localization and non-co-localization, respectively. Bar indicates 50 μm (A–F). [file Image1.JPEG]
